# α-Galactosidase and Sucrose-Kinase Relationships in a Bi-functional AgaSK Enzyme Produced by the Human Gut Symbiont *Ruminococcus gnavus* E1

**DOI:** 10.3389/fmicb.2020.579521

**Published:** 2020-11-12

**Authors:** Mickael Lafond, Alexandra S. Tauzin, Laetitia Bruel, Elisabeth Laville, Vincent Lombard, Jérémy Esque, Isabelle André, Nicolas Vidal, Frédérique Pompeo, Nathalie Quinson, Josette Perrier, Michel Fons, Gabrielle Potocki-Veronese, Thierry Giardina

**Affiliations:** ^1^Aix-Marseille Université, CNRS, Centrale Marseille, iSm2, Marseille, France; ^2^TBI, Université de Toulouse, CNRS, INRAE, INSA, Toulouse, France; ^3^CNRS, Aix-Marseille Université, AFMB, Marseille, France; ^4^Yelen Analytics, Aix-Marseille Université, ICR, Marseille, France; ^5^Aix-Marseille Université, CNRS, IMM, LCB, Marseille, France; ^6^Aix-Marseille Université, CNRS, IMM, BIP, Marseille, France

**Keywords:** raffinose oligosaccharide family, sucrose, α-galactosidase, sucrose-kinase, human gut microbiome, GH36 family

## Abstract

Plant α-galactosides belonging to the raffinose family oligosaccharides (RFOs) and considered as prebiotics, are commonly degraded by α-galactosidases produced by the human gut microbiome. In this environment, the *Ruminococcus gnavus* E1 symbiont–well-known for various benefit–is able to produce an original *Rg*AgaSK bifunctional enzyme. This enzyme contains an hydrolytic α-galactosidase domain linked to an ATP dependent extra-domain, specifically involved in the α-galactoside hydrolysis and the phosphorylation of the glucose, respectively. However, the multi-modular relationships between both catalytic domains remained hitherto unexplored and has been, consequently, herein investigated. Biochemical characterization of heterologously expressed enzymes either in full-form or in separated domains revealed similar kinetic parameters. These results were supported by molecular modeling studies performed on the whole enzyme in complex with different RFOs. Further enzymatic analysis associated with kinetic degradation of various substrates followed by high pressure anionic exchange chromatography revealed that catalytic efficiency decreased as the number of D-galactosyl moieties branched onto the oligosaccharide increased, suggesting a preference of *Rg*AgaSK for RFO’s short chains. A wide prevalence and abundance study on a human metagenomic library showed a high prevalence of the *Rg*AgaSK encoding gene whatever the health status of the individuals. Finally, phylogeny and synteny studies suggested a limited spread by horizontal transfer of the clusters’ containing *Rg*AgaSK to only few species of Firmicutes, highlighting the importance of these undispersed tandem activities in the human gut microbiome.

## Introduction

Raffinose family oligosaccharides (RFOs) are mainly found in the seeds of vegetables. From a structural point of view, RFOs are alpha-galactosyl derivatives of sucrose, and the family mainly contains raffinose (i.e., one galactosyl unit attached to sucrose), stachyose (i.e., two D-galactosyl units attached to sucrose) and verbascose (i.e., three D-galactosyl units attached to sucrose) with a degree of polymerization (DP) from 3 to 5, respectively. In some plants, RFOs are formed by up to 15 galactosyl residues (Bachmann et al., 1994). No human enzyme is able to degrade these compounds. Dietary RFOs pass through the stomach and the small intestine without major changes to reach the colon almost unchanged before being fermented or degraded by anaerobic commensal bacteria (Martínez-Villaluenga et al., 2008). During decades, RFOs were considered to be antinutritional factors, as their consumption in large quantities leaded to negative effects such as abdominal pains, flatulence, bloating and diarrhea, mainly due to their fermentation in the colon (Martínez-Villaluenga et al., 2008). Nowadays, the vision of the RFOs is changing thanks to the in-depth studies carried out in the last decade and they are now considered as prebiotics (Collins and Reid, 2016) as their consumption at the right dose could promote the growth of beneficial bacteria including Bifidobacteria and Lactobacilli (Ejby et al., 2016; Collins et al., 2017; Zartl et al., 2018). In addition, *in vitro* studies have also shown that the presence of raffinose can reduce the growth of pathogens (Shoaf et al., 2006). Therefore, in the highly competitive ecosystem constituted by the gastrointestinal tract, RFOs represent a selective advantage for organisms capable of metabolizing them to the detriment of others, and for the host health.

In the CAZy database^1^, the bacterial enzymes responsible for the degradation of RFOs, α-galactosidases, are classified into the glycoside hydrolase (GH) families 4, 27, 36, 57, 97, and 110, but mainly gathered within the families GH27 and GH36 (Lombard et al., 2014). Over the last 15 years, the structures of several α-galactosidases have been determined within the GH27, GH36, and GH97 families (Fujimoto et al., 2003, 2009; Garman and Garboczi, 2004; Golubev et al., 2004; JCSG., 2005, 2013; Comfort et al., 2007; Lieberman, 2009; Okuyama et al., 2009; Fernández-Leiro et al., 2010; Guce et al., 2010; Vorobiev et al., 2010; Bruel et al., 2011; Fredslund et al., 2011; Merceron et al., 2012; Yu et al., 2014; Adamson et al., 2016; Kikuchi et al., 2017; Kytidou et al., 2018; Hobbs et al., 2019; Rowland et al., 2019). The α-galactosidases from GH27 and GH36 families share a common catalytic domain characterized by a (β/α)_8_-barrel folding type and a C-terminal domain composed of β-sheets. In contrast, α-galactosidases in the GH36 family have an N-terminal domain that is missing in GH27 family members (Comfort et al., 2007).

Remarkably, a bifunctional enzyme belonging to the GH36 family, the α-(1,6)-galactosidase/sucrose kinase (*Rg*AgaSK) naturally produced by *Ruminococcus gnavus* E1, a human gut commensal isolated from healthy human feces (Ramare et al., 1993), was identified (Bruel et al., 2011). The *RgAgaSK* encoding gene, found highly transcribed *in vivo*, belongs to the *Rgaga1* polysaccharide utilization locus (PUL)–suggested to be involved in RFOs metabolization–which includes one transcriptional regulator, three ABC transporter components and one sucrose 6^*Fructose*^-phosphate phosphorylase (*Rg*SPP) encoding genes (Bruel et al., 2011; Tauzin et al., 2019). In addition, the study of the *Rgaga1* PUL organization highlighted a high synteny with loci from *Blautia hansenii* DSM 20583, *Eubacterium rectale* ATCC 33656 and to a lesser extent *Clostridium* sp. L2-50 (Tauzin et al., 2019). Interestingly, *Rg*AgaSK consists of two domains: one closely related to α-galactosidases GH36 family and the other one containing a nucleotide-binding motif (Walker A motif) (Bruel et al., 2011). The bi-functional *Rg*AgaSK enzyme has shown a high sequence identity (i.e., more than 90%) with enzymes from *B. hansenii* DSM 20583 and *E. rectale* ATCC 33656, while the locus from *Clostidium* sp. L2-50 harbors separated α-galactosidase and kinase encoding genes, the latter being completely absent in other related loci from *Lactobacillales* (Tauzin et al., 2019). From a biochemical point of view, *Rg*AgaSK showed its ability to hydrolyze soluble α-galactosides derived from sucrose (e.g., raffinose), and also to use ATP as co-substrate to phosphorylate specifically sucrose on the C6 position of glucose residue generating sucrose-6^*Glucose*^-phosphate (S6P). In addition, *Rg*SPP, from the same PUL, showed a selective activity on sucrose 6^*Fructose*^-phosphate (S6FP) acting both in phosphorolysis releasing α-D-glucose-1-phosphate (G1P) and α-D-fructose-6-phosphate (F6P), and in reverse phosphorolysis from G1P and F6P to S6FP. These results bring out an original glycolytic pathway from a well-known gut bacterium due to a unique PUL involved in RFO metabolic pathways reinforcing the crucial role of the kinase domain (Bruel et al., 2011; Tauzin et al., 2019).

Here, we investigate the relationships between the kinase and the α-galactosidase GH36 domains using biochemical, enzymatic and molecular modeling approaches. A phylogenetic analysis of the whole GH36 family was constructed to gain insight into the α-galactosidase evolution–especially for the *Rg*Aga module–while a synteny study was carried out to examine the spread of the *Rgaga1* PUL. Finally, the abundance and the prevalence of the *RgAgaSK* gene were quantified in the human gut metagenome to assess its importance in healthy or pathological contexts.

## Results

### Expression and Purification of the Recombinant RgAgaSK and Its Truncated Variants

**FIGURE 1:**
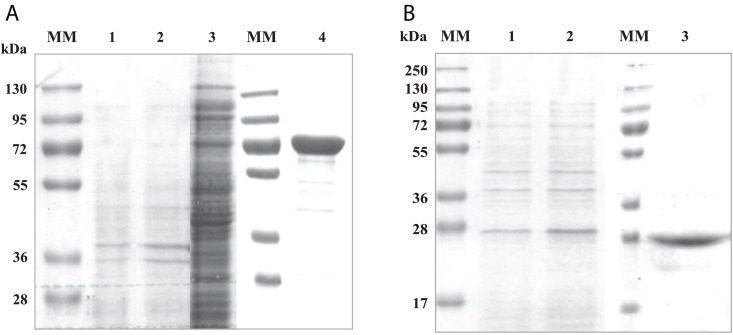
SDS-PAGE analysis of recombinant *Rg*Aga **(A)** and *Rg*SK **(B)** expressed in *Escherichia coli* BL21 cells. **(A)** (1) Samples from cell extracts of *E. coli* BL21 *pOPINE* after induction, (2) cell extracts of *E. coli* BL21 *pOPINE:rgaga* before induction, (3) cell extracts of *E. coli* BL21 *pOPINE:rgaga* after induction (4) purified fraction of *Rg*Aga after elution from affinity chromatography and (MM) molecular weight markers. **(B)** (1) Samples from cell extracts of *E. coli* BL21 *pOPINE:rgsk* before induction, (2) cell extracts of *E. coli* BL21 *pOPINE:rgsk* after induction, (3) purified fraction of *Rg*SK after elution from affinity chromatography and (MM) molecular weight markers.

To investigate the interactions between both domains of the *Rg*AgaSK enzyme, we generated two variants consisting of each domain individually expressed in *Escherichia coli* BL21 cells, the N-terminal domain corresponding to the α-galactosidase domain (*Rg*Aga, from Ala-1 to Lys-720) and the C-terminal domain corresponding to the kinase domain (*Rg*SK, from Arg-724 to Gln-935), respectively. The proteins were purified by Ni^2+^ affinity chromatography. The purified recombinant proteins showed major bands on SDS-PAGE at 80 and 25 kDa, respectively (Figures 1A,B, respectively). The molecular masses of these recombinant proteins were consistent with their theoretical molecular mass (81,245 and 23,898 Da, respectively). In each case, up to 10 mg.L^–1^ of pure recombinant proteins were obtained.

**FIGURE 2:**
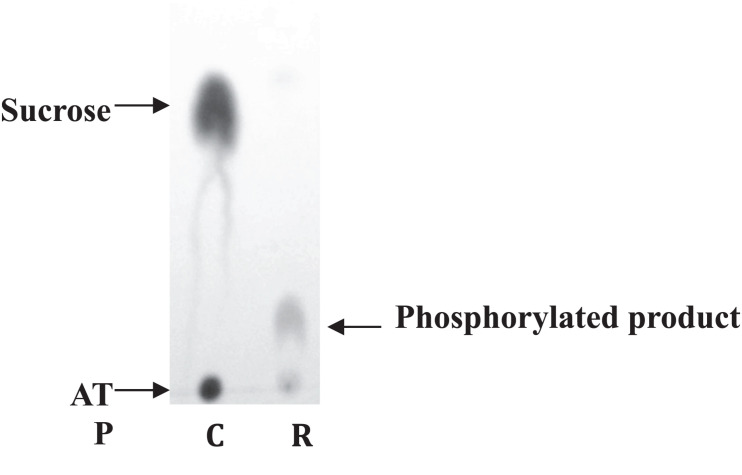
Sucrose kinase activity analysis of *Rg*SK by TLC. Sucrose and ATP (R) were incubated together for 30 min at 30°C in presence of *Rg*SK. The control (C) corresponded to the phosphorylation reaction in the presence of *Rg*SK previously denatured.

To ensure the functionality of the isolated kinase domain, a kinase activity assay has been used in presence of sucrose and ATP, as previously described for *Rg*AgaSK (Bruel et al., 2011; Figure 2). The migration profile of the phosphorylation reaction using *Rg*SK showed the production of a phosphorylated-sucrose, product demonstrating the ability of *Rg*SK to catalyze a phosphorylation reaction.

### Stability of RgAgaSK and RgSK

**FIGURE 3:**
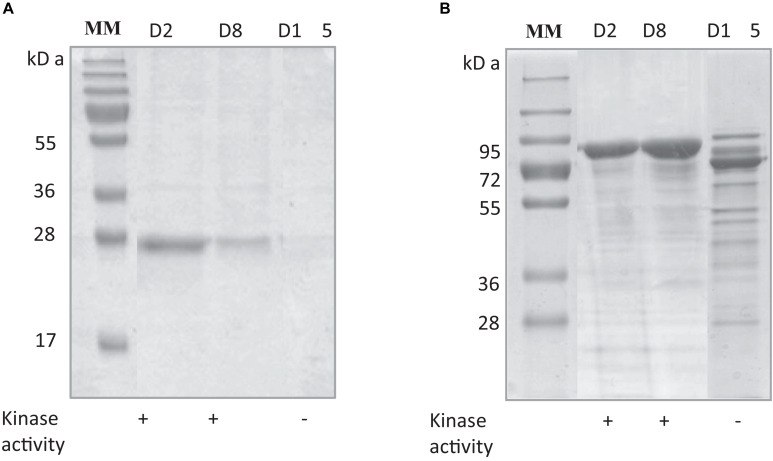
Comparison of protein degradation and sucrose kinase activity over time of **(A)** *Rg*SK and **(B)** *Rg*AgaSK. The proteins were stored at +4°C in HEPES buffer 50 mM at pH 7.0. The degradation of **(A)** *Rg*SK and **(B)** *Rg*AgaSK was evaluated by protein separation using SDS-PAGE and their respective kinase activity was evaluated on TLC, whose results are referenced in the tables under the gels. (+) means presence of sucrose kinase activity, whereas (–) correspond to absence of sucrose kinase activity.

The stability of *Rg*AgaSK and *Rg*SK during storage at 4°C was tested, and protein degradation and sucrose kinase activity were monitored over time: 2 (D2), 8 (D8), and 15 (D15) days (Figure 3). The evaluation of the protein degradation on SDS-PAGE showed that *Rg*SK was stable up to 8 days and completely degraded after 15 days, whereas *Rg*AgaSK results in a partial degradation with the main band corresponding to the *Rg*Aga module alone (∼80 kDa).

### RgAgaSK and RgAga: α-Galactosidase Activity Comparison on a Synthetic Substrate

The effects of pH and temperature on the enzyme stability were investigated for both recombinant enzymes, and no significant difference was found between *Rg*AgaSK (whole enzyme) and *Rg*Aga (α-galactosidase domain). Indeed, enzymes displayed an optimum activity in the pH range from 5.0 to 7.0, whereas the activity was lost under pH 3.0 and over pH 8.0 (Figure S1). At pH 6.0, the optimum temperature was 42°C for both enzymes, and the activity drastically decreased above 55°C (Figure S1). The hydrolysis of *p*NPGal as substrate confirmed the α-exo-acting activity of the *Rg*Aga domain. Moreover, the kinetic parameters in optimum conditions showed that the absence of the kinase domain in *Rg*Aga did not alter the affinity (*K*_*m*_) with *p*NPGal substrate (1.2 ± 0.2 mM *versus* 1.3 ± 0,2 mM for *Rg*AgaSK). The *V*_*max*_ and the catalytic efficiency (*k*_*cat*_/*K*_*m*_) were the same order of magnitude (Table 1).

**TABLE 1:** Physicochemical and kinetic parameters for *Rg*AgaSK and *Rg*Aga using *p*NPGal as substrate.

	*Rg*AgaSK	*Rg*Aga
**Physicochemical parameters**		
T° optimum (°C)	42	42
pH optimum	6.0	6.0
**Kinetic parameters**		
V_*max*_ (nmol.min^–1^)	178.7 ± 6.9	236.4 ± 15.8
K_*m*_ (mM)	1.3 ± 0.2	1.2 ± 0.2
k_*cat*_ (s^–1^)	625.8 ± 24.0	827.0 ± 52.0
k_*cat*_/K_*m*_ (s^–1^.M^–1^)	4.81.10^5^	6.89.10^5^

*The pH and temperature values correspond to the average of two different assays and the kinetic parameters values correspond to the average of three different assays.*

### RgAgaSK and RgAga: α-Galactosidase Activity Comparison on Natural Substrates

**FIGURE 4:**
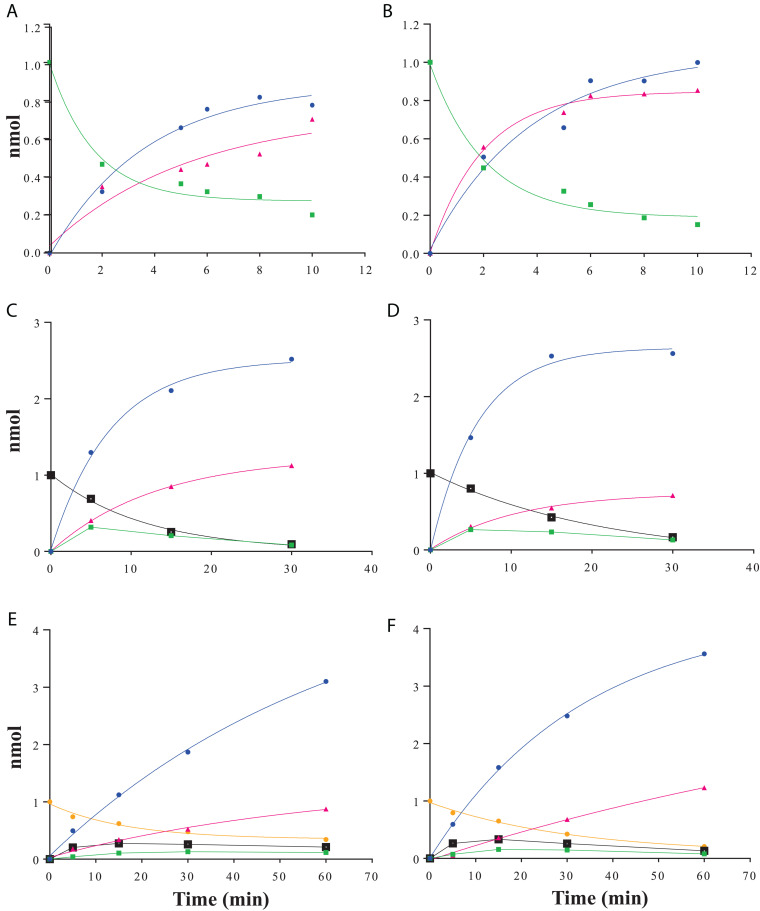
RFOs hydrolysis kinetics using HPAEC-PAD analysis. Raffinose hydrolysis by *Rg*AgaSK **(A)** and *Rg*Aga **(B)**; stachyose hydrolysis by *Rg*AgaSK **(C)** and *Rg*Aga **(D)** and verbascose hydrolysis by *Rg*AgaSK **(E)** and *Rg*Aga **(F)**. Galactose appears in blue, sucrose in pink, raffinose in green, stachyose in black, and verbascose in yellow. Recombinant proteins (25 nM) were incubated at 42°C with 100 μM of stachyose. The values correspond to the averages of two isolated tests.

Raffinose family oligosaccharides hydrolysis is usually measured using a global method (galactose oxidase or carbohydrate reducing end) which does not allow determining the hydrolysis kinetic when one of the products formed is itself a substrate. In order to analyze the α-galactosidase activity of *Rg*AgaSK and *Rg*Aga toward natural substrates derived from plants [i.e., RFOs including raffinose (DP3), stachyose (DP4), and verbascose (DP5)], we followed RFOs hydrolysis using anionic exchange chromatography coupled with a pulsed amperometric detection (HPAEC-PAD) (Figure 4). Both *Rg*AgaSK and *Rg*Aga showed similar hydrolysis profiles of raffinose (Figures 4A,B). In each case, 50% of the raffinose were consumed in 2 min reaching 80% after about 10 min. In parallel, the equimolar increase of galactose was a good indicator of the reliability of the method used (see blue and green line points in Figures 4A,B). Otherwise as observed in Figures 4C,D, stachyose was completely hydrolyzed after 30 min, and the profiles were similar whatever the protein modularity considered, i.e., with or without kinase domain. During the first 5 min, the hydrolysis of stachyose was concomitant with the production of raffinose, sucrose, and galactose. This result means that for two stachyose molecules in the medium, one was completely degraded (two galactoses and one sucrose) whereas the second was partially (one galactose and one raffinose), leading to a molar ratio of 3:1 for galactose and raffinose/sucrose, respectively. However, after 5 min, raffinose did not accumulate contrary to the sucrose, meaning that the majority of raffinose was completely degraded (two galactoses and one sucrose). Finally, for verbascose as for stachyose, no accumulation of intermediate products (i.e., stachyose and raffinose) was observed while the sucrose considered as a final product increased all over the hydrolysis reaction (Figures 4E,F).

**FIGURE 5:**
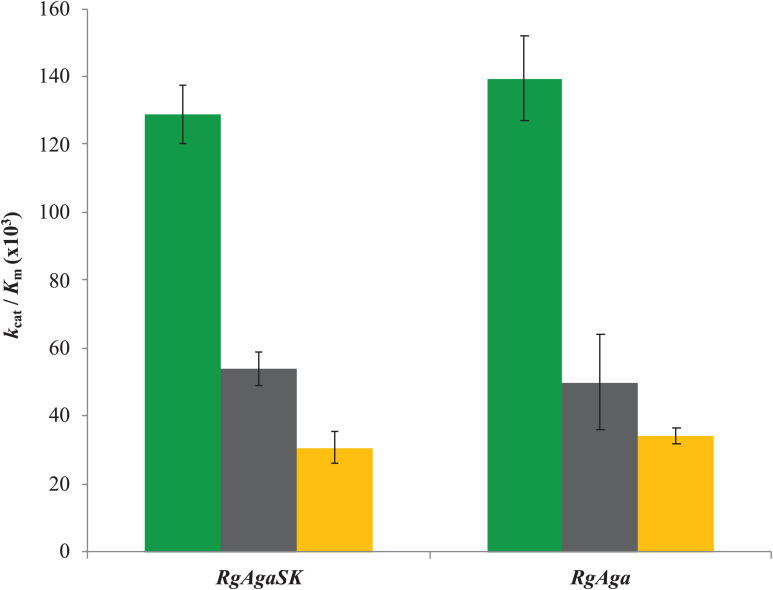
Catalytic efficiencies of *Rg*AgaSK and *Rg*Aga toward different RFOs. Raffinose appears in green, stachyose in black, and verbascose in yellow. Catalytic efficiencies were calculated from results obtained between 0 and 10 min for raffinose hydrolysis and between 0 and 5 min for stachyose and verbascose hydrolysis. The values corresponded to the average of two different assays.

Subsequently, the catalytic efficiencies (*k*_*cat*_/*K*_*m*_) of *Rg*AgaSK and *Rg*Aga toward the different RFOs were determined using the Matsui equation (Matsui et al., 1991; Figure 5). No substantial difference was observed between the two enzymes whatever the substrate used, i.e., raffinose, stachyose, and verbascose. Interestingly, for both enzymes, the catalytic efficiency is dependent on the substrate length. Indeed, this value decreases when the substrate length – the number of galactosyl residues – increases (i.e., DP3 to DP5), as shown in Figure 5.

### RgAgaSK and RgSK: Hydrolytic and Kinase Activities Comparison on Natural Substrates

In order to investigate the influence of the α-galactosidase domain on kinase activity, the kinetic parameters of *Rg*AgaSK and *Rg*SK using sucrose as substrate were analyzed and compared.

#### Kinetic Parameters of Kinase Activity

**FIGURE 6:**
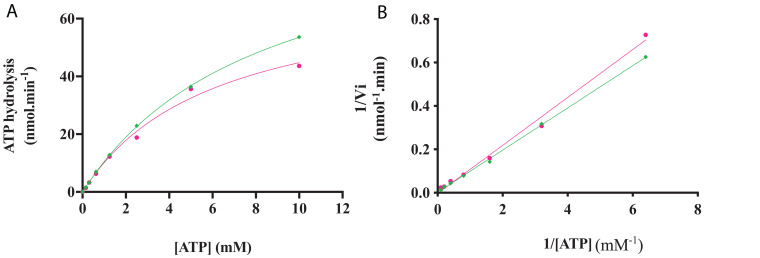
*Rg*AgaSK and *Rg*SK kinetics toward ATP. The phosphorylation rate of ATP by *Rg*AgaSK (in green) and *Rg*SK (in pink) was evaluated by using the representation of Michaelis-Menten **(A)** and Lineweaver Burk **(B)** plot. The proteins (1.3 μM) were incubated with varying concentrations of ATP ranging from 0 to 10 mM for 3 min at 30°C and pH 8.0. The values correspond to the average of three different assays.

The kinase activity of *Rg*AgaSK and *Rg*SK was evaluated following ATP consumption using bioluminescence assay. Under these basal conditions, different enzymatic activity analyses were performed for both *Rg*AgaSK and *Rg*SK using different concentrations of ATP from 0 to 10 mM (Figure 6). Regarding ATP hydrolysis, the results showed that in both cases the enzymes presented Michaelian kinetics (Figure 6). The Michaelis constant (*K*_*m*_) and the catalytic constant (*k*_*cat*_) were similar as those of *Rg*AgaSK (8.4 ± 1.9 mM and 38.1 ± 4.8 s^–1^, respectively), and for *Rg*SK (6.3 ± 1.8 mM and 27.9 ± 4.3 s^–1^, respectively). In addition, the catalytic efficiencies (*k*_*cat*_/*K*_*m*_) calculated for *Rg*AgaSK and *Rg*SK were 4.53.10^3^ and 4.42.10^3^ s^–1^.M^–1^, respectively (Table 2). To conclude, as observed above, the absence of the α-galactosidase domain does not seem to affect the kinase activity.

**TABLE 2:** Comparison of *Rg*AgaSK and *Rg*SK ATP hydrolysis kinetic parameters and phosphorylation kinetic parameters using sucrose as substrate.

	*Rg*AgaSK	*Rg*SK
**ATP hydrolysis**		
V_*max*_ (nmol.min^–1^)	98.3 ± 12.5	71.9 ± 11.2
K_*m*_ (mM)	8.4 ± 1.9	6.3 ± 1.8
k_*cat*_ (s^–1^)	38.1 ± 4.8	27.9 ± 4.3
k_*cat*_/K_*m*_ (s^–1^.M^–1^)	4.53.10^3^	4.42.10^3^
**Sucrose phosphorylation**		
V_*max*_ (nmol.min^–1^)	9.9 ± 2.7	7.2 ± 0.6
K_*m*_ (mM)	3.1 ± 1.5	1.0 ± 0.2
k_*cat*_ (s^–1^)	3.3 ± 0.9	2.4 ± 0.2
k_*cat*_/K_*m*_ (s^–1^.M^–1^)	1.06.10^3^	2.40.10^3^

*The values correspond to the average of three different assays.*

#### Kinetic Parameters of Phosphorylation Activity

**FIGURE 7:**
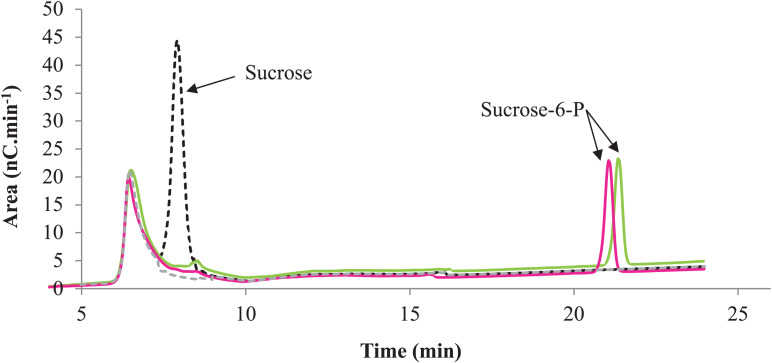
Phosphorylation profile of sucrose by *Rg*AgaSK and *Rg*SK. HPAEC-PAD chromatograms superimposition with *Rg*AgaSK reaction appearing in green, with *Rg*SK appearing in pink. The reaction control (black dash line) was the reaction with denatured enzyme, and the buffer control (gray dash line) was the buffer only without substrates and enzymes.

**FIGURE 8:**
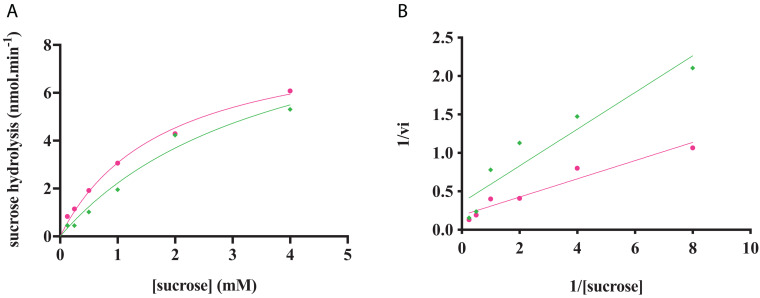
*Rg*AgaSK and *Rg*SK kinetics toward sucrose. The phosphorylation rate of sucrose by *Rg*AgaSK (in green) and *Rg*SK (in pink) was evaluated by using the representation of Michaelis-Menten **(A)** and Lineweaver Burk **(B)** plot. The proteins (50 nM) were incubated with varying concentrations of sucrose ranging from 0 to 4 mM for 5 min at 30°C. The values are the average of three different assays.

**FIGURE 9:**
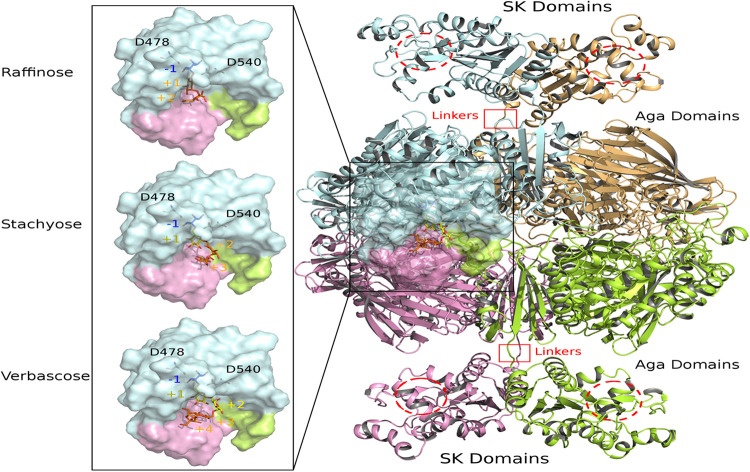
View of modeled quaternary assembly of *Rg*AgaSK-RFO complexes. The tetrameric *Rg*AgaSK structure is shown as carton on the right. Each subunit is highlighted according to a specific color (pale cyan, bright orange, lemon, and pink). SK Domains are located on the top and the bottom of the complex with putative active sites indicated with red dashed circles. Aga domains are viewed as an assembly in the middle, connected to the SK domains by linkers highlighted by red squares. The active site of one Aga subunit is shown as transparent surface. A zoom on this region is displayed on the left with three complexed ligands (raffinose, stachyose, and verbascose) docked independently in Aga active site. For reference, the catalytic residues are shown in ball & sticks at subsite –1. The galactosyl bound in the subsite –1 is colored in blue whereas the sucrose moiety at the end chain is colored in orange. Additional galactosyl units within stachyose and verbascose are colored in yellow.

The characterization of the phosphorylation activity on sucrose by *Rg*AgaSK and *Rg*SK was assessed by HPAEC-PAD. Analysis of the phosphorylation reaction profiles for each form showed the formation of a single product eluted at 22 min (Figure 7) and identified as S6P by mass spectrometric analysis (Figure S2). Then, in order to determine and compare the kinetic parameters of *Rg*AgaSK and *Rg*SK for sucrose phosphorylation reaction, enzymatic activity analyses were performed in initial rate conditions with different sucrose concentrations (Figure 8). The results obtained showed that both *Rg*AgaSK and *Rg*SK had a Michaelian sucrose phosphorylation kinetics with a Michaelis constant (*K*_*m*_) and a catalytic constant (*k*_*cat*_) for *Rg*AgaSK of 3.1 ± 1.5 mM and 3.3 ± 0.9 s^–1^ and for *Rg*SK of 1.0 ± 0.2 mM and 2.4 ± 0.2 s^–1^, respectively (Figure 8). The analysis of specificity constants allowed to calculate a similar affinity for sucrose between the two proteins but a two times faster phosphorylation rate for *Rg*SK. The calculation of the catalytic efficiency (*k*_*cat*_/*K*_*m*_), which reflects the specificity of the enzyme with respect to its substrate, makes it possible to compare the activities of the two proteins between them with 1.06.10^3^ and 2.40. 10^3^ s^–1^.M^–1^ for *Rg*AgaSK and *Rg*SK, respectively (Table 2). Thus, conversely to the results observed above between *Rg*AgaSK and *Rg*SK where the SK domain has no impact to the α-galactosidase activity, here the absence of the α-galactosidase domain seems slightly affecting the kinase activity leading to an increase of the phosphorylation activity. This observation could be explained by the topology of the domain interactions where the *Rg*SK domain could not interact with the *Rg*Aga active site due to the length of the linker, rather than *Rg*SK seems more dependent of the linker flexibility and of the *Rg*Aga domain movement on the bottom (see Figure 9 below).

### Modeling of Enzyme Assemblies and Complexes With RFOs

The 3D model of *Rg*AgaSK was built using the 3D X-ray structure structure of α-galactosidase domain in its tetrameric assembly [PDB: 2YFN; (Bruel et al., 2011)], which was then fused to the *Rg*SK domain modeled by threading techniques due to the lack of structural templates covering the full sequence. The spatial constraints imposed by the quaternary organization of *Rg*Aga and the C-terminal orientation of the four chains led to the introduction of the *Rg*SK domains oriented toward the exterior of the tetrameric assembly (Figure 9). The proposed 3D model of *Rg*AgaSK suggests that interface between *Rg*SK domains involves a network of stabilizing interactions. It also reveals that such *Rg*SK domains are not located in the proximity of *Rg*Aga active sites, although conformational rearrangements of the linkers could bring closer *Rg*Aga and *Rg*SK domains without creating interferences in cross-talking of active sites as suggested previously.

Detailed inspection of the 3D organization of *Rg*Aga domains reveals that substrate binding pocket is formed by loop regions of the catalytic (β/α)_8_-barrel and also from N- and C-terminal regions of the two adjacent subunits (Figure 9), as earlier reported (Bruel et al., 2011). Catalytic dyad, formed by the nucleophile Asp478 and the acid/base Asp540, is buried at the bottom of *Rg*Aga active site pocket (subsite −1), properly oriented with respect to the anomeric center of the galactosyl residue at the non-reducing end of the RFOs (see blue sticks in Figure 9). Molecular surface representation of *Rg*Aga domains also reveals an active site of about 12Å depth with subsites +2, +3 rather exposed to the solvent whereas subsite −1 is buried within the protein (Figure 9). α-galactosidases from *Geobacillus stearothermophilus* (*Gs*AgaA and *Gs*AgaB), which both present 46% of sequence identity with the *R. gnavus* E1 α-galactosidase domain, also share a highly similar quaternary organization (Merceron et al., 2012).

**FIGURE 10:**
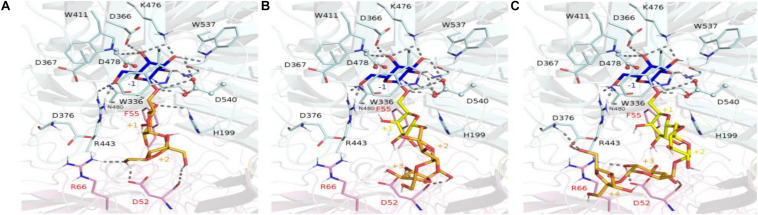
Molecular view of RFOs docked in *Rg*Aga active site. The interacting amino acid residues and the ligands, i.e., raffinose **(A)**, stachyose **(B)**, and verbascose **(C)**, are shown in sticks and colored with the same color code as in Figure 9. Dashed lines represent the potential hydrogen bonds between any ligand with their surrounding residues. The binding subsites are labeled as –1, +1, +2, +3, and +4.

The crystallographic structure of the inactivated *Gs*AgaA was earlier determined in complex with raffinose and stachyose bound in subsites −1, +1, +2, and +3, enabling a detailed mapping of the network of stabilizing interactions (Merceron et al., 2012). This network of interactions reported to stabilize sugars bound at subsites −1, +1, and +2 is found to be maintained in complexes of *Rg*Aga with docked RFOs. While binding interactions are definitely more extensive at subsite −1 involving amino acid residues Trp336, Asp366, Trp411, Arg443, Lys476, Trp537, positive subsites progressively involve fewer interactions with bound sugars mostly involving amino acid residues from adjacent subunit such as Asp52, Phe55, Arg66 (Figure 10). All amino acid residues interacting with RFOs are highly conserved with the exception of His199 from subsite +1.

### Phylogenetic Analysis of the GH36 Family

**FIGURE 11:**
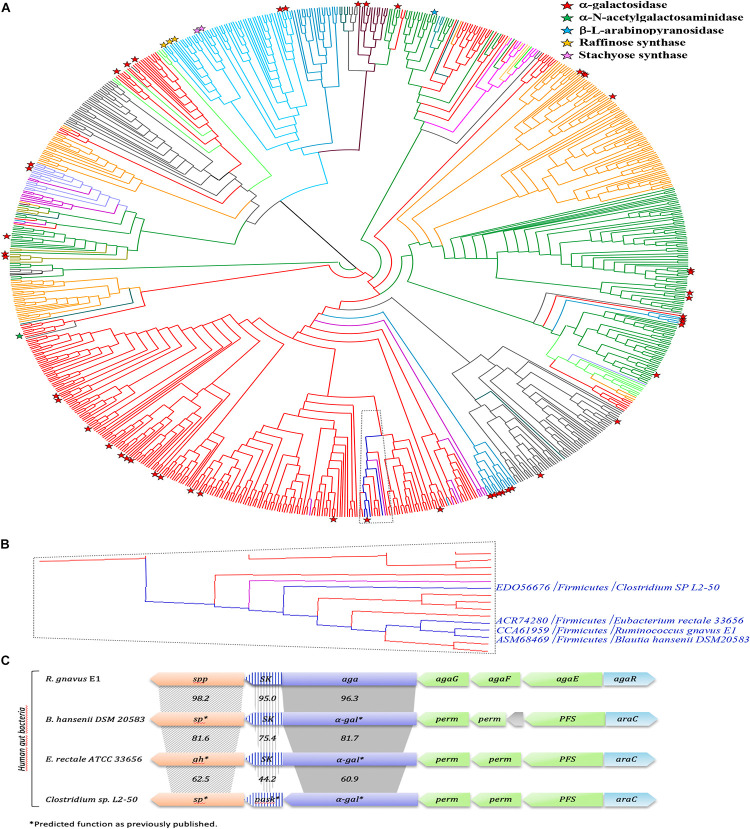
Phylogenetic study of the GH36 family and *Rg*AgaSK genomic environment. **(A)** A Phylogenic tree of the GH36 family was constructed including all the GH36 referenced in the CAZy database at 11/06/2019. Members sharing full synteny with *RgAgaSK* are indicated in deep blue. The tree has been colored according to the phylogenetic classification of the organisms. Indeed, Firmicutes appeared in red, Bacteroidetes in gray, Proteobacteria in orange, Actinobacteria in deep green, Acidobacteria in light green, Eukaryota in light blue, Fungi in medium blue, Archaebacteria in deep violet, Spirochaetes in light violet, Thermotogae in light purple, Verrucomicrobia in water-green, Deinococcus in kaki, and Chloroflexi in pink. Characterized enzymes are labeled with a red star for their α-galactosidase activity, a green star for their α-N-acetylgalactosaminidase activity, a blue star for their β-L-arabinopyranosidase activity, an orange star for their raffinose synthase activity and a pink star for their stachyose synthase activity. **(B)** Zoom of the clade which includes *RgAgaSK* and members sharing full synteny with *RgAgaSK* (in deep blue) and other sequences from human gut bacteria. **(C)** Genomic environment of the *RgAgaSK* encoding gene. The values indicate the percentage of identities between the encoding genes according to their modularity i.e., *kinase*, α*-gal* and *spp* or *sp*.

In order to gain more insight into the phylogenetic diversity of the GH36 family, a phylogenetic tree has been constructed, using the 843 sequences extracted from the CAZy database (Figure 11A; Lombard et al., 2014). GH36 enzymes are distributed in diverse phylogenetic groups from different ecosystems like soil, marine or human gut microbiome and covering the three super-kingdoms. The present phylogenetic and taxonomic analysis reveals five main clades containing characterized α-galactosidase enzymes (i.e., *Firmicutes*, *Actinobacteria*, *Proteobacteria*, *Bacteroidetes*, and *Eukaryota*). The distribution of the 75 characterized GH36 enzymes is globally well spread over the phylogenetic tree, with an α-galactosidase activity found for most of them. Nevertheless, specificities are not restricted to galactosidases. Indeed, α-N-acetylgalactosaminidase, β-L-arabinopyranosidase, raffinose synthase or even stachyose synthase activities are also identified mainly in *Eukaryota* clade. Therefore, we observed that *Rg*AgaSK belongs to a *Firmicutes* clade constituted by almost only bacterium from the intestinal microbiome (e.g., *Blautia*, *Clostridium*, *Eubacterium*, *Roseburia*, *Coprococcus*) (Figure 11B). Interestingly, the SK domain appeared linked with α-galactosidase domain only for some of species found in this *Firmicutes* clade including *Rg*AgaSK (Figure 11C).

As previously mentioned, the *RgAgaSK* gene is located into the *Rgaga1* PUL described by Bruel et al. (2011), and detailed by Tauzin et al. (2019) (Figure 11C). To figure out the accurate role of such Gram positive PUL in the human gut microbiome, we performed a synteny study based on all the prokaryotic strains containing GH13_18 and GH36 neighborhood enzymes coupled with a deep mining of *R. gnavus* E1 genome, that led us to propose a new model of sucrose, S6^*F*^P and RFOs metabolic pathways (Figure 11C; Tauzin et al., 2019). As represented in this last figure, the loci alignment shows (i) only four syntenies with high conservation of this usual gram positive PUL organization model (i.e., with *B. hansenii*, *E. rectale* and with a lesser extent *C. spL2-50*), (ii) high identities between α*-gal* or *sk* genes (i.e., more than 60%), and (iii) high identities between *spp* genes (i.e., more than 62%) which are always in downstream position of those encoding the bi-functional kinase and α-galactosidase enzymes. Nevertheless, the GH36 sequences comparative analysis showed a synteny limited to these four loci, and that not all sequences found in the same clade as *Rg*AgaSK, or even in the whole phylogenetic tree, are included in similar PULs (Figure 11). This observation actually suggests that there is no common ancestor, but that dissemination has occurred by horizontal evolution to only a few species within the human digestive tract.

### Prevalence and Abundance in the Human Gut Microbiome

**FIGURE 12:**
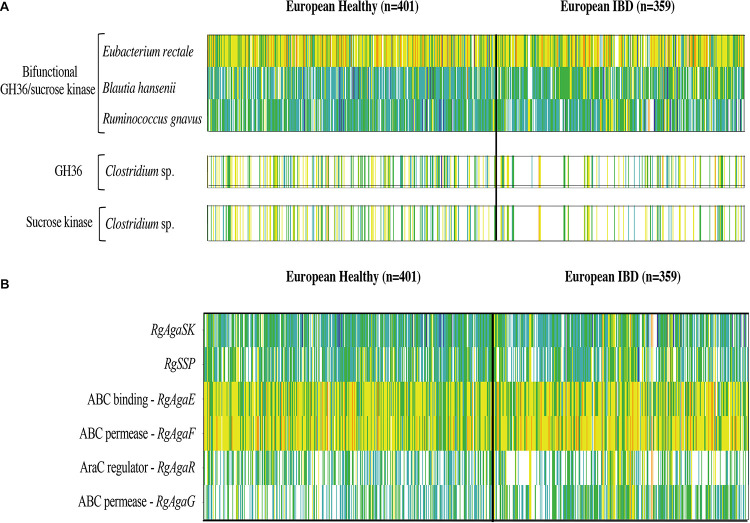
Abundance and prevalence of the GH36 and sucrose kinase encoding genes targeted in this study **(A)**, and of the genes of the *R. gnavus Rgaga1* locus **(B)** in the human gut microbiome of European healthy and IBD affected individuals. Gene abundance in the gut metagenome of 760 European subjects is represented by a color scale: white, not detected; blue, turquoise, green, yellow, and orange, increasing abundance with a 10-fold change between colors.

Then, we searched the microbial gene richness of the four known GH36 associated to a kinase whether in the form of a bifunctional protein or in the form of two distinct proteins in the microbiome of 760 European subjects, by recovering prevalence and abundance of the homologous sequences in the frequency matrix of genes (MetaHIT Consortium, Li et al., 2014; Figure 12A). The four GH36s found in syntenic PULs belong to gram-positive Firmicutes listed in the most frequent components of the human indigenous intestinal microbiome (MetaHIT Consortium, Qin et al., 2010). The bimodular GH36 of *R. gnavus* E1, *B. hansenii* DSM 20583 and *E. rectale* ATCC 33656 (accession numbers CCA61959, ASM68469, and ACR74280, respectively), are highly prevalent whatever the medical status of the subjects. The gene of *E. rectale* was the most abundant. Besides, the separated GH36 and SK genes of *Clostridium sp.* L2-50 (accession numbers EDO56676 and WP_008399817.1, respectively) present the same richness profiles. They are less prevalent in IBD subjects than in healthy ones.

In addition, we examined the prevalence and abundance of the genes of *R. gnavus Rgaga1* locus previously described in Bruel et al. (2011) and in Tauzin et al. (2019). We found six homologous genes in the human gut catalog. The prevalence and abundance profiles of the six genes of the cluster are different, with a higher abundance of *AgaE* and *AgaF* genes involved in substrate binding and transport (Figure 12B).

## Discussion

Here, the function of each domains of the *Rg*AgaSK, *Rg*Aga, and *Rg*SK, have been investigated. The two domains *Rg*Aga and *Rg*SK have been cloned and expressed separately in *E. coli* cells. Analysis of the physico-chemical properties of the α-galactosidase domain revealed no difference with the whole protein, whereas according to the kinetic parameters, *Rg*Aga appears slightly more efficient for *p*NPGal hydrolysis compared to *Rg*AgaSK. However, these differences were not observed with the tested RFOs: raffinose, stachyose and verbascose. Thus, the *Rg*Aga activity on natural substrates was not significantly disturbed by the presence nor absence of the kinase domain, leading us to suggest that *p*NPGal had probably a non-specific interaction with the kinase domain.

The hydrolysis of RFOs (raffinose, stachyose, and verbascose) by *Rg*AgaSK and *Rg*Aga was analyzed by following substrate consumption and products release using HPAEC-PAD analysis. This method was essential, as the substrates of interest are α-galactosylated derivatives of sucrose which means that some of the products, resulting from the hydrolysis of RFOs by α-galactosidase, are themselves substrates. Indeed, in most of the studies found in the literature, hydrolysis is followed by a global method that doses all of the released galactose residues–using galactose oxidase (Tauzin et al., 2019) or carbohydrate reducing ends using 3,5-dinitrosalicylic acid (McLauchlan et al., 1999)–without knowing exactly from which molecule present in the medium the galactose was derived. For stachyose and verbascose, chromatogram analysis showed no product corresponding to galactobio-oligosaccharides [(galactose-α(1,6)-galactose)_*n*_], confirming the α-exo-acting specificity of the *Rg*AgaSK. All kinetics obtained show that during RFOs hydrolysis, there was no accumulation of intermediate molecules, i.e., raffinose or stachyose, suggesting that the hydrolysis of the different RFOs was done randomly and the degradation was complete until obtention of sucrose as end product. Analysis of the hydrolysis profiles for each RFO showed no significant difference between the kinetics observed with *Rg*AgaSK or *Rg*Aga, suggesting that the presence of the kinase domain did not interfere the hydrolysis process for natural substrates. The analysis of the literature data reinforces this conclusion with similar *k*_*cat*_/*K*_*m*_ values for raffinose with *Rg*AgaSK (12.9.10^4^ s^–1^.M^–1^) as for *Thermotoga maritima* (18.7.10^4^ s^–1^.M^–1^) and *Thermotoga neapolitana* (10.6.10^4^ s^–1^.M^–1^) in which none kinase domain is associated to the α-galactosidase catalytic module (King et al., 1998; Miller et al., 2001).

Overall, our proposed 3D modeling supports experimental results that indicate that the presence of *Rg*SK domains does not affect the activity of the *Rg*Aga enzyme on RFOs. Nevertheless, for both enzyme modularities–with or without the *Rg*SK domain–and depending on the RFOs assayed, catalytic efficiencies decreased as the number of galactosyl residues attached to the oligosaccharide increased. This observation could be explained by the 3D model which shows a high exposition to the surface of the longer RFO leading to a flexibility, and probably less affinity so either *K*_*m*_ increases due to this flexibility, either the *k*_*cat*_ decreases because the flexibility affects the good orientation for the catalysis.

Considering now the *Rg*AgaSK and *Rg*SK modularities, their characterization on ATP hydrolysis ability was evaluated by bioluminescence. In both cases, the analysis of affinity (*K*_*m*_) toward ATP and hydrolysis catalytic efficiency (*k*_*cat*_/*K*_*m*_) did not show significant differences, suggesting that the presence of the *Rg*Aga domain on the one hand does not interfere with ATP binding as affinities were nearly similar and, on the other hand, that its position did not affect accessibility to the ATP binding domain resulting in a comparable hydrolysis rate.

The phosphorylation activity of sucrose by *Rg*AgaSK and *Rg*SK was then assessed by HPAEC-PAD analysis, which allowed separating the different carbohydrates according to their degree of ionization. Phosphate groups being highly ionizable, the interaction of phosphorylated sucrose with the resin is therefore be stronger and the retention time longer than that of sucrose. Analysis of the affinity of both recombinant proteins toward sucrose showed no significant differences, suggesting that the presence of the *Rg*Aga domain does not affect sucrose binding in the active site of the kinase domain. However, the phosphorylation of sucrose in terms catalytic efficiency (*k*_*cat*_/*K*_*m*_) has almost doubled between *Rg*AgaSK (1.06.10^3^ s^–1^.M^–1^) and *Rg*SK (2.40.10^3^ s^–1^.M^–1^), which suggests that the presence of the *Rg*Aga domain slightly affects the phosphorylation activity of the kinase module. This hypothesis can be supported by the 3D model. Indeed, according to the topology of the assembly and the orientation of each domain and active site, the probability to affect the SK active site is higher.

Independently, the TLC analysis of sucrose phosphorylation in the presence of ATP revealed that protein degradation is associated with a loss of kinase activity after 15 days of storage for both *Rg*SK and *Rg*AgaSK. These observations showed (i) functionality of the kinase domain alone, which suggested that the functional folding of the ATP binding site is done independently of the presence of the α-galactosidase domain, and (ii) proved that *Rg*AgaSK instability is related to its kinase domain.

Our phylogenic study reveals that the 75 characterized GH36s so far are spread all over the tree covering relatively well the family. Some of the members were found in the *Firmicutes* groups, known as the second main phyla in the human gut microbiome (Gentile and Weir, 2018). It is noteworthy that *Rg*AgaSK is the only characterized member of a clade comprising solely bacteria from the intestinal microbiome (e.g., *Blautia*, *Clostridium*, *Eubacterium*, *Roseburia*, *Coprococcus*).

Nevertheless, in a previous study focusing on the characterization of the GH13 (*Rg*SPP) from the *Rgaga1* locus, we observed that the whole *Rgaga1* locus (see Figure 11C) showed a high synteny with loci from *B. hansenii*, *E. rectale* and *C. sp. L2-50* (Tauzin et al., 2019). This gene organization was not present for other GH36s clustered in the same clade or even in the whole family. This observation leads to the conclusion that the *Rgaga1* PUL dissemination has occurred by horizontal evolution and was restricted to only few species within the human digestive tract.

We searched for homolog to the genes of the *Rgaga1* locus in the human gut metagenomic gene catalog and retrieved their occurrence frequency values from the gene frequency table in 760 European subjects.

The genes encoding for the bimodular kinase/GH36 from *R. gnavus* E1, *B. hansenii* DSM 20583, and *E. rectale* ATCC 33656 are highly prevalent (Figure 12A). When both modules are encoded by separated genes, as found in *Clostridium sp. L2-50*, both genes display similar prevalence/abundance profiles suggesting a close association of these genes in microorganisms and probably synergistic activity in the metabolic pathway as a bimodular enzyme. In contrast, the six genes of the *Rgaga1* locus have very different abundance profiles, especially a higher abundance of *AgaE* and *AgaF* genes involved in substrate binding and transport (Figure 12B). This suggests that the locus is not conserved in many gut bacterial genomes, and that each of its components could operate independently following different metabolic paths from different substrates (raffinose, sucrose, and S6FP) as presented in Tauzin et al. (2019).

Finally, we noticed that the kinase and GH36 genes of *Clostridium sp. L2-50* are less prevalent in the IBD subjects which is in accordance with the finding of Manichanh (2006) showing that the fecal microbiota of patients with Crohn’s disease contains a markedly reduced diversity of Firmicutes of which some *Clostridium* species are less prevalent and abundant. Although some Clostridia groups possess pathogenic species, most of them have commensal relationships with the host, playing an important role in the metabolic welfare of epithelial cells of the colon by releasing butyrate as an end-product of fermentation of monosaccharides (Lopetuso et al., 2013).

To conclude, we confirmed that the *Rg*AgaSK is perfectly tuned to face both α-galactosidase and kinase activities. Indeed, the *Rgaga1*-like PULs code for two proteins (i.e., *Rg*SPP and *Rg*AgaSK), yielding three complementary enzyme activities involved in the metabolism of sucrose and its derivatives (RFOs) and providing an evolutionary advantage to certain human gut bacteria to colonize their competitive environment.

## Materials and Methods

### Cloning, Expression and Purification of RgAgaSK, RgAga, and RgSK

*Rg*AgaSK was produced as previously described by Bruel et al. (2011). The *rgaga* and *rgsk* genes were amplified from plasmid containing full *RgAgaSK* gene with PCR reaction primers (Table S1). Recombinant proteins, with C-terminal His^6^-tag, were synthesized from *E. coli* BL21 cells (DE3) grown in LB broth, containing 50 mg.L^–1^ ampicillin and 1 mM IPTG, during an overnight culture at 23°C. Bacterial lysis were carried out with “Cell Disruptor” (Constant System LTD) in the binding buffer for affinity chromatography (50 mM HEPES at pH 7.7, 150 mM NaCl and 10 mM imidazole) at 1.37 kbar. After centrifugation at 10,000 × *g* during 20 min at 4°C, the soluble fractions were loaded onto a Ni-NTA column and the recombinant proteins were eluted with 125 mM imidazole in 50 mM HEPES at pH 7.7 and 150 mM NaCl. Fractions containing proteins were pooled, dialyzed against 50 mM HEPES buffer at pH 7.0 and concentrated with 10,000 molecular weight cutoff (MWCO) filter using Amicon Ultra centrifugal filter devices. The concentrated proteins were injected in HiPrep 16/60 Sephacryl^TM^ S-300, using 50 mM HEPES at pH 7.0 and 200 mM NaCl with a flow rate of 1 mL.min^–1^ and purified to near homogeneity (>90%). The active fractions were pooled and dialyzed against HEPES buffer 50 mM at pH 7.0. The purity of the protein was checked by SDS-PAGE (12%, Laemmli, 1970). The protein concentration was determined using the Bio-Rad protein assay kit with bovine serum albumin as standard or measuring the absorbance at 280 nm. The molar extinction coefficient used were: 142,740, 119,695, and 23,045 M^–1^.cm^–1^ for *Rg*AgaSK, *Rg*Aga, and *Rg*SK, respectively.

### Galactosidase Assay

Galactosidase activity was measured using the synthetic substrate *p*NPGal. The enzyme (48 nM) was incubated with 2 mM *p*NPGal in McIlvaine’s buffer (100 mM citric acid, 200 mM Na_2_HPO_4_, pH 6.0) in 96 microtiter wells (200 μl), and the increase in absorbance at 405 nm was monitored for 10 min at 42°C in a microplate reader [KRL test (Spiral patent); Kirial International]. One unit of enzyme activity was defined as the amount of protein that released 1 μmol of *p*NP/min at 42°C and pH 6.0. The extinction coefficient for the *p*NP under this condition was 1,582.72 M^–1^.cm^–1^.

### Temperature, pH, and Kinetic Parameters

The optimal pH was determined on *p*NPGal (20 mM) in McIlvaine’s buffer in a pH range of 3.0 to 8.0. The optimal temperature was determined at temperatures ranging from 10 to 70°C. For determination of the apparent Michaelis-Menten constants, the initial velocities of the enzymes were measured at 42°C in McIlvaine’s buffer at pH 6.0, with *p*NPGal concentrations ranging from 0.078 to 10 mM. The kinetic parameters were estimated using non-linear regression with GraphPad Prism software (GraphPad Prism version 3.00 for Windows 95; GraphPad Software, San Diego, CA, United States).

### Bioluminescence Kinase Activity Assay

The characterization of ATP hydrolysis activity by *Rg*AgaSK and *Rg*SK was evaluated by bioluminescence, which allowed quantity determination of ATP present in the reaction assay using luciferin/luciferase reagent according to manufacturer’s instructions (YELEN Analytics, France) and luminometer reader (Tecan, France) described in Razafimanjato et al. (2011). Thus, for *Rg*AgaSK and *Rg*SK the initial rate conditions were determined at pH 8.0 and 30°C, in saturated sucrose condition (5 mM), with 1.3 μM of enzyme for three minutes. Under these conditions, different enzymatic activity analyses were performed for both *Rg*AgaSK and *Rg*SK using different concentrations of ATP (0 to 10 mM) and maintaining sucrose concentration to 5 mM.

### RFO Product Hydrolysis Assay

The hydrolysis of RFOs was performed under optimal temperature and pH conditions with 25 nM of enzyme and 100 μM of substrate. The reaction was stopped by addition of 130 mM NaOH. The samples were centrifuged for 3 min at 13,000 × *g* and injected (20 μL) on the HPAEC system (ICS-5000^®^, Thermo Fisher Scientific, Courtaboeuf, France). Elution was achieved by a linear gradient of sodium acetate (0 to 130 mM) in 130 mM NaOH for 19 min at a flow rate of 1 mL.min^–1^. The elution of mono-, di- and oligosaccharides was monitored by an electrochemical detector (Pulse Amperometric Detection, PAD). The calibration curves ranging from 0 to 100 μM were designed with the different substrates used and products formed (galactose, sucrose, raffinose, stachyose, and verbascose), which allowed their release or consumption to be quantified using the Chromeleon^®^ software (Dionex). All analyses were duplicated.

To evaluate α-galactosidase activity on RFOs, the initial slopes of the regression lines are used. The catalytic efficiency (*k*_*cat*_/*K*_*m*_) of the reactions is calculated using the Matsui equation: [E]^∗^(*k*_*cat*_/*K*_*m*_)^∗^t = ln([S_0_]/[S_*t*_]), with[E] the enzyme concentration used in nM, (*k*_*cat*_/*K*_*m*_) the catalytic efficiency in min^–1^.M^–1^, t time in minutes, [S0] and [St] substrate concentration at time 0 min and time t in nM, respectively (Matsui et al., 1991).

### 3D Modeling of RgAgaSK

The full sequence of *RgAgaSK* from *R. gnavus* E1 was extracted from UniProt (G4T4R7). The α-galactosidase domain comprising the first 719 amino acids at the N-terminal of *Rg*AgaSK was determined by X-ray crystallography (PDB-ID: 2YFN, Bruel et al., 2011) with a resolution of 1.45Å. The kinase domain, corresponding to the last 215 amino acid residues of the C-terminal domain of *Rg*AgaSK, was modeled by threading techniques using the I-TASSER webserver (Zhang, 2008; Roy et al., 2010; Yang et al., 2015) with a C-score of −0.05 and using mainly as template the crystallographic structure of *Thermus thermophilus* HB8 uridine kinase (PDB code: 3ASY, Tomoike et al., 2011) Modeler 9.19 (Webb and Sali, 2016) was subsequently used to merge the *Rg*Aga and *Rg*SK domains into *Rg*AgaSK and build the tetrameric assembly. For each step, ten models were generated using automodel class and fixing *Rg*Aga domain coordinates. Finally, the models with lowest DOPE score (Shen and Sali, 2006) were chosen for modeling *Rg*AgaSK-RFOs complexes.

### 3D Modeling of RgAgaSK-RFO Complexes

The binding mode of three distinct RFOs in *Rg*AgaSK active site was then investigated. Three complexes were built, *Rg*AgaSK-RAF (complex with raffinose), *Rg*AgaSK-STACH (complex with stachyose) and *Rg*AgaSK-VERB (complex with verbascose). *Rg*AgaSK-RAF and *Rg*AgaSK-STACH complexes were built after superimposition onto the crystal structure of *G. stearothermophilus* α-galactosidase co-crystallized in complex with raffinose (PDB-ID: 4FNT) and with stachyose (PDB-ID: 4FNU). The complex with verbascose (*Rg*AgaSK-VERB) was built using GLYCAM_06j-1 Force Field (Kirschner et al., 2008) and the program tleap of AMBERTOOLS17 (Case et al., 2017). Then, geometry of verbascose ligand was fitted onto stachyose binding mode. Finally, all complexes were minimized in vacuum with a cut-off of 99Å and 500 steps of steepest descent then 500 steps of conjugated gradient. All graphics were prepared using PyMOL 1.7 (Schrödinger).

### Phylogenetic Analysis of the GH36 Family

A phylogenetic analysis of the GH36 family was performed on CAZy release (November 06th, 2019) considering the bounded modules of 6,125 sequences in the public database. Signal sequences and additional modules were removed to isolate the catalytic modules for bioinformatics analysis. The amino acid sequences were aligned with MAFFT v7.271 (Katoh, 2005) and clustered above 95% identities using CD-HIT version 4.6 reducing the number of sequences considered to 843. We used the option–maxiterate 1000 and–localpair to obtain high accuracy where–maxiterate # indicates the maximum number of iterative refinement and localpair forces local pairwise alignment. To generate the tree, we built a matrix of maximum likelihood distances based on model of substitution LG (Le and Gascuel, 2008). Finally, the phylogeny reconstruction tree was performed using BIONJ (Gascuel, 1997). Evolutionary tree construction was conducted using Dendroscope software (Huson et al., 2007).

Cluster analysis was based on the neighbor-joining method with the closely related bacterium *R. gnavus* E1 as the out-group root. Synteny blocks were analyzed by the MaGE platform that allows the comparison of CDS predicted from genomic DNA of *R. gnavus* E1 to those predicted from genomic DNA present in the bases PkGDB (Prokaryotic Genome DataBase) and NCBI RefSeq (collection of Raw sequences from whole genome sequencing). Beyond a simple sequence comparison, this interface allows to analyze the synteny between two chromosomes. Only, strictly conserved synteny has been considered here and a multiple sequence alignment was produced using Muscle program (Edgar, 2004) to evaluate the sequence identities percentage between closely modules i.e., between α-galactosidases, SKs, or SPPs.

The prevalence and abundance of the GH36/sucrose kinase genes in the human fecal microbiome were determined by searching for the homolog sequences in the translated catalog of 9.9 million reference genes using BLASTP, *E*-value = 0, identity ≥ 90% (MetaHIT Consortium, Li et al., 2014). The gene richness in the human gut was assessed by recovering the occurrence frequency data of the homolog sequences identified in the catalog from the gene frequency table in 760 European subjects consisting of two cohorts, one of healthy individuals and the other of individuals in remission from inflammatory bowel disease (Li et al., 2014).

## Data Availability Statement

The ORFs and protein sequences of RgAgaSK protein were previously deposited in GenBank under the accession number FQ790379.1 and CCA61959.1, respectively (Bruel et al., 2011).

## Author Contributions

ML and AT were involved in data acquisition, data interpretation, and manuscript writing. LB was involved in biochemical and enzymatic analysis and data interpretation. EL and GP-V was involved the prevalence analysis. VL was involved in the sequences extraction from CAZy database and phylogenic analysis. NV was involved in the bioluminescence assay. NQ, MF, and JP were involved in the data interpretation. JE and IA planned and performed molecular modeling studies and contributed to manuscript writing. FP was involved in data acquisition. TG was the coordinator of the study and was involved in the manuscript writing and editing. All authors read and approved the final manuscript.

## Conflict of Interest

The authors declare that the research was conducted in the absence of any commercial or financial relationships that could be construed as a potential conflict of interest.

## Footnotes

1 www.cazy.org
