# Supplementary material for: α-Galactosidase and Sucrose-Kinase Relationships in a Bi-functional AgaSK Enzyme Produced by the Human Gut Symbiont Ruminococcus gnavus E1
Source: Front Microbiol. 2020 Nov 12;11:579521. doi: 10.3389/fmicb.2020.579521 (PMC7688924; doi:10.3389/fmicb.2020.579521)
Supplement: Supplementary file 1 [file Data_Sheet_1.PDF]

Supplementary Material

α-galactosidase and sucrose-kinase relationships in a bi-functional AgaSK enzyme produced by the human gut symbiont *Ruminococcus gnavus* E1

Mickael Lafond[1#], Alexandra S. Tauzin[2#], Laetitia Bruel[1], Elisabeth Laville[2], Vincent Lombard[3], Jérémy Esque[2], Isabelle André[2], Nicolas Vidal[4], Frédérique Pompeo[5], Nathalie Quinson[1], Josette Perrier[1], Gabrielle Potocki-Veronese[2] and Thierry Giardina[1*]

[1] Aix-Marseille Univ, CNRS, Centrale Marseille, iSm2, Marseille, France

[2] TBI, Université de Toulouse, CNRS, INRAE, INSA, F-31400 Toulouse, France

[3] CNRS, Aix-Marseille Univ, AFMB, Marseille, France

[4] Yelen Analytics, Aix-Marseille Univ, ICR, Marseille, France

[5] Aix-Marseille Univ, CNRS, IMM, LCB, Marseille, France

# These authors contributed equally to this work.

*** Correspondence:** Thierry Giardina: thierry.giardina@univ-amu.fr

Table S1: Primers used for *Rgaga*, *Rgsk* and *RgagaSK* cloning

***RgagaSK* cloning primers**	
pOPINErgagaskS	AGGAGATATACCATGGCAATTATATACAATCCA
pOPINErgagaskAS	GTGATGGTGATGTTTCTGTTCATAAACACTTCC
***Rgaga* cloning primers**	
pOPINErgagaskS	AGGAGATATACCATGGCAATTATATACAATCCA
pOPINErgagaAS	GTGATGGTGATGTTTCAGTTCGGTAAAGGC
***sk* cloning primers**	
pOPINErgskS	AGGAGATATACCATGGCGGGAAGATTATATG
pOPINErgagaskAS	GTGATGGTGATGTTTCTGTTCATAAACACTTCC

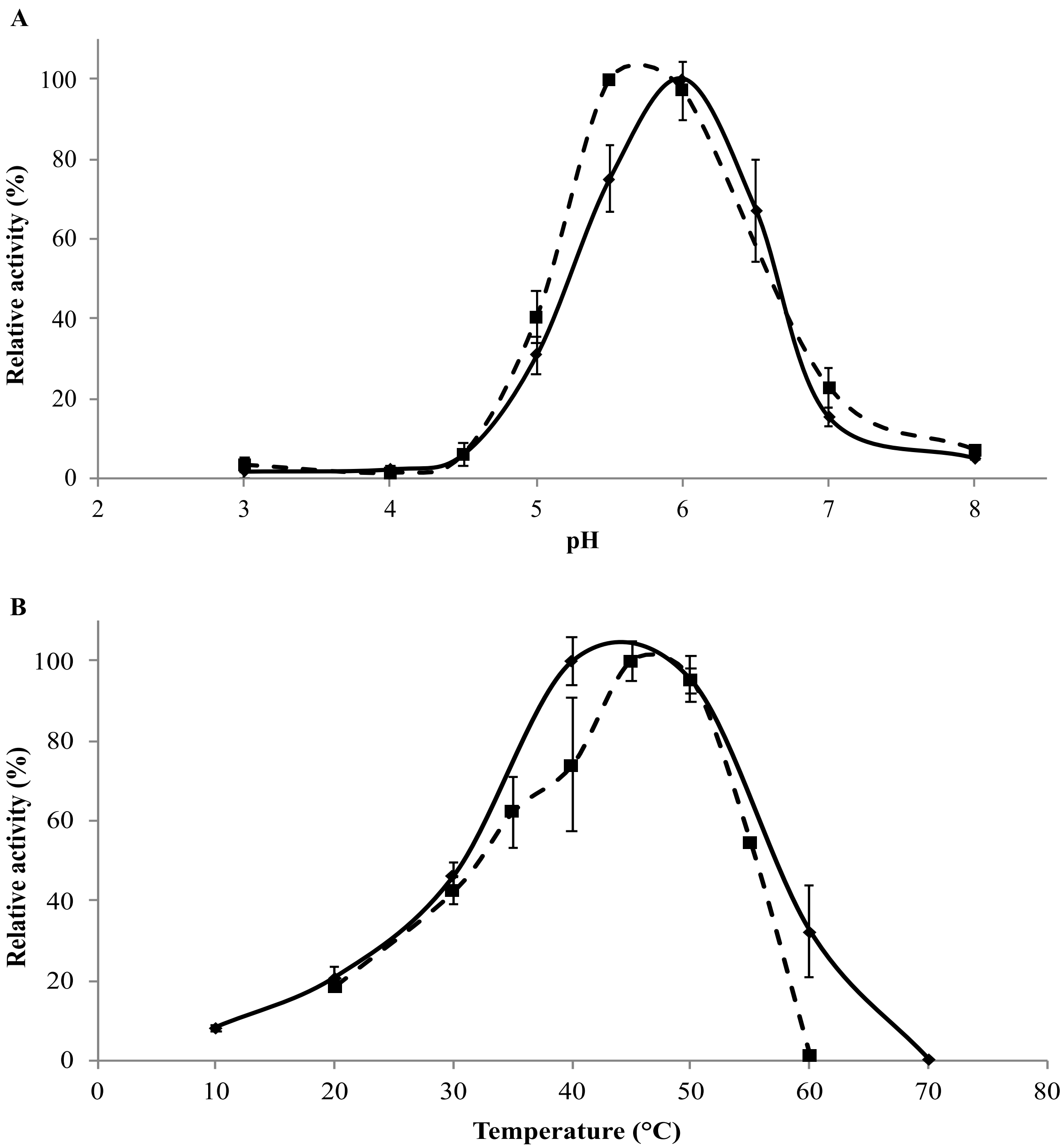

Figure S1: Optimum pH and temperature profiles. Optimum pH has been determined from 3.0 to 8.0 (**A**), whereas optimum temperature between 10 to 70°C, (**B**). *Rg*AgaSK appears in full line and *Rg*Aga appears in dashed line.

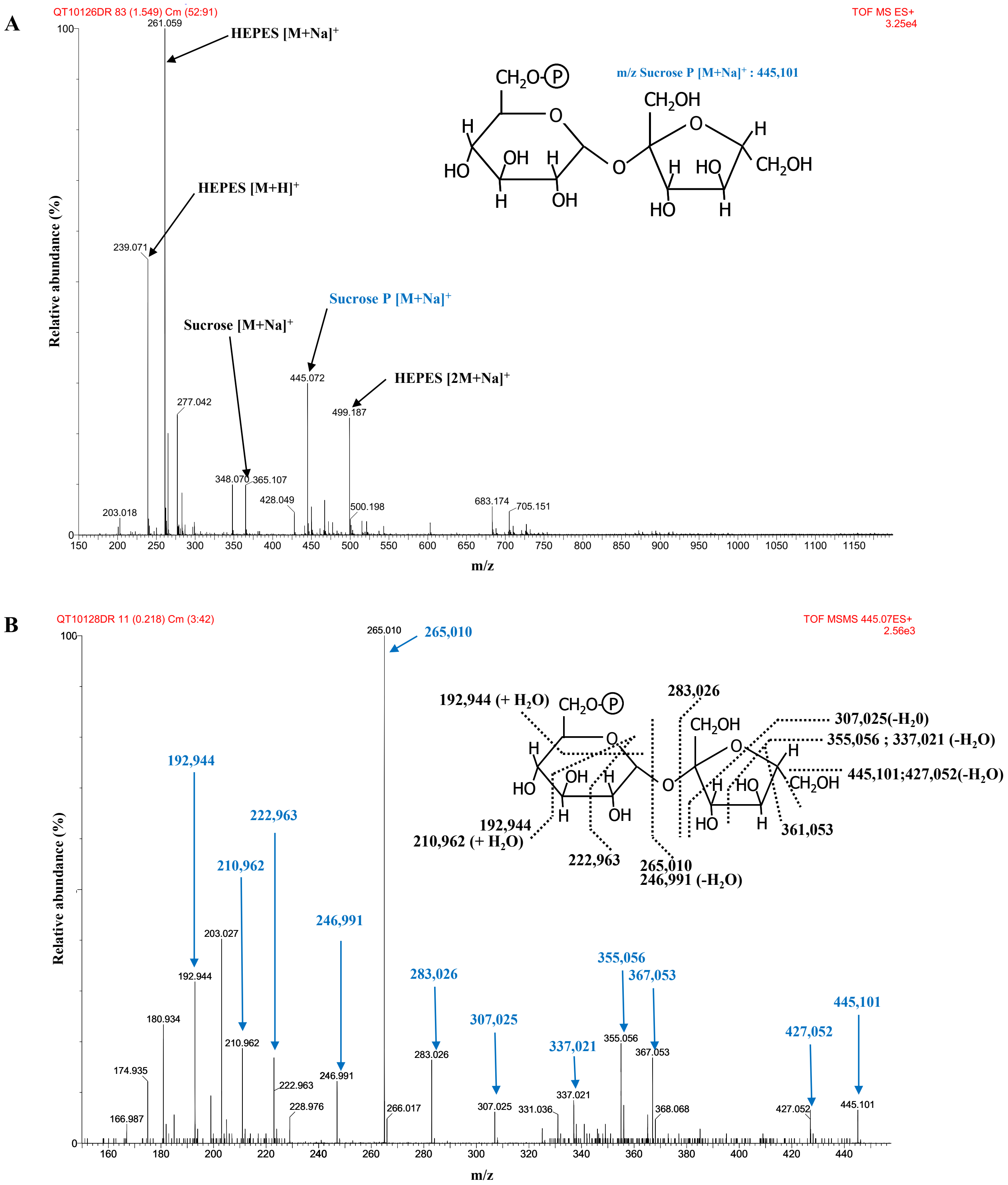

Figure S2: Mass spectrometry analysis of the *Rg*AgaSK reaction products, sucrose-6-phosphate. Deconvoluted ESI-Q-Tof MS (A) and MS/MS (B) spectrum of the sucrose-6-phosphate with a m/z = 445,101.

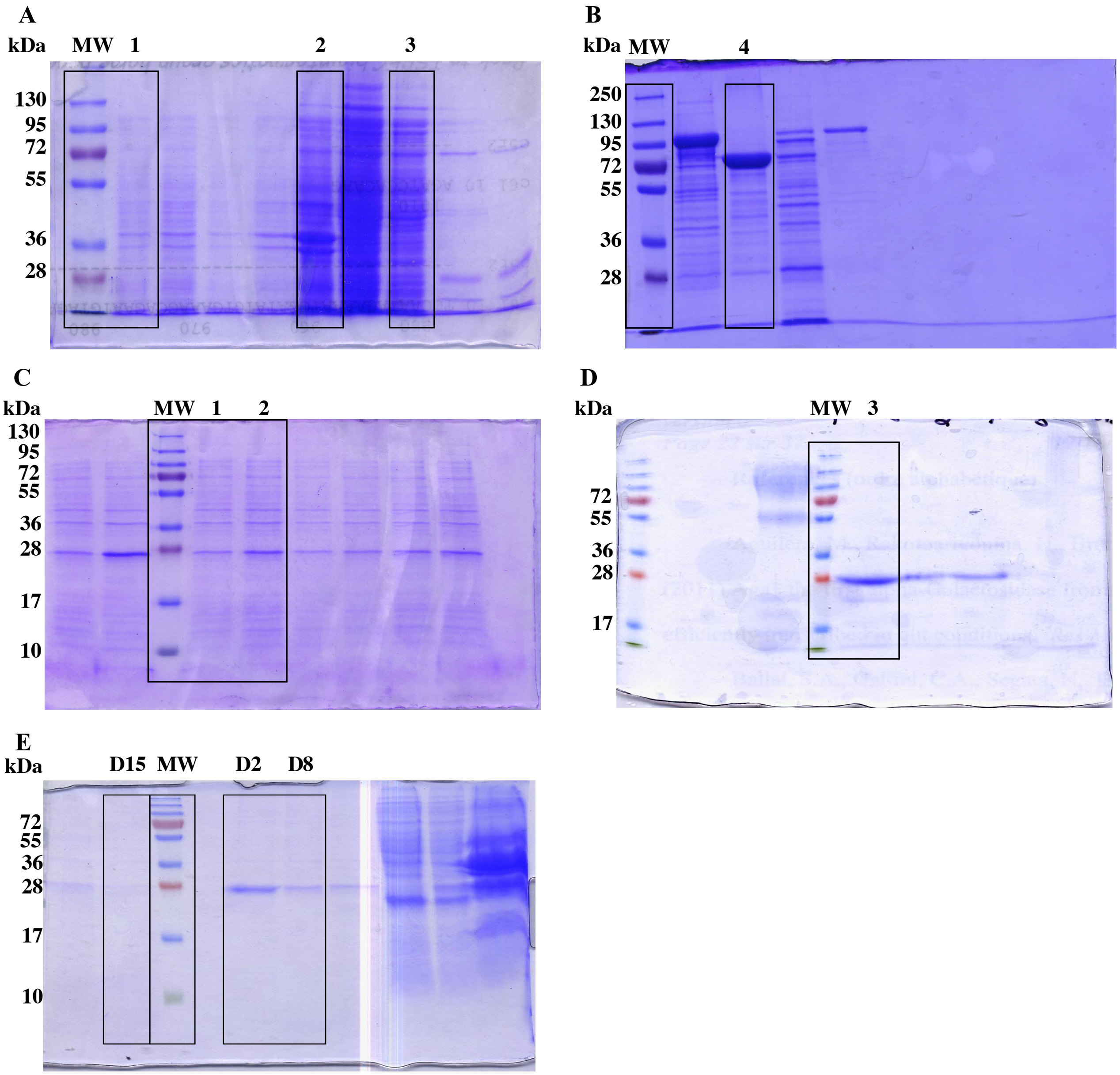

Figure S3: Full SDS-PAGE corresponding to those showed in Figures 1A (A & B), 1B (C & D), and 3A (E).
